# Medical Department of the University of Buffalo

**Published:** 1856-09

**Authors:** 


*Medical Department of the University of Buffalo.*—As the period for the beginning of the lecture-term approaches, it becomes evident that there is to be a large increase in the number of students in this institution. To this end many circumstances contribute. The steady policy of the school has been to offer no clap-trap inducements to students. In the face of an active competition as to lecture fees, it has uniformly placed its claim to support on its advantages, and not its cheapness. When other schools have lowered their fees, it has raised them. When others have permitted graduations within the period of study fixed by the American Medical Association, it has uniformly lived up to the letter and spirit of its requirements. It has made its examinations public, and has endeavored to win the confidence of the profession by protecting its honor and its interests. This is, we acknowledge, a slow policy, but it is a truthful one, and sure to meet its reward.

Other and more incidental facts contribute to its success. The present season has been fruitful in changes in our medical schools. Resignations, owing partly to the small remuneration of professorships have been frequent. The vacant places, from the same cause, find no applicants to fill them. Chairs go begging for occupants, and in many instances it has been found impossible to supply them, and the circulars of the schools are sent out with blanks attached to their most important professorships.

In other and more prosperous schools, the faculty has been weakened by the transfer of their more distinguished men to the great eastern schools. It is becoming the policy of these institutions to watch the rising greatness of western men, and, when it has become assured, to transplant them in the east. Several instances of this kind have occurred during the present vacation, and the slow, but sure, process of medical centralization is steadily going on. Its results must be ultimately injurious, but the stubborn fact is there, and we of the west are compelled to recognize it.

But while all these changes have been going on, the Buffalo school changes only for the better. While others lose by resignation, it retains all its old teachers; while others are suffering from the drafts of eastern schools, it gains in number and talent; and while others cannot fill their vacancies, it calls well known and widely recognized teachers to its aid.

Says the Buffalo Commercial Advertiser of Aug. 28th: "Its faculty is now one of the largest in the country. The recall of Prof. Austin Flint, as Professor of Clinical Medicine and Pathology, has been a subject of warm congratulation among its friends, while the subsequent recall of Prof. M. Moore, of Rochester, and his acceptance of the chair of Surgical Anatomy and Surgical Pathology, is another strong element of success. At a time when vacancies in other schools are filled with the greatest difficulty, the College has been particularly fortunate in securing such men to fill, not vacancies, but chairs created especially for them, and not taught as distinct branches in any other school.

" We are happy to learn that there is now a flattering prospect of a large increase in the number of students, and it is confidently expected that the term will open in October with a larger class than any for several years. The opportunities afforded by the institution for instruction by the bedside, have always been considered one of its best features. No other institution, even in the great eastern cities, has as fine *available* means of teaching the science of disease by the bedside. The Hospital of the Sisters of Charity, not a stone's throw from the College building, is open to all the students without any extra fee."

